# Novel point cloud registration approach for noninvasive patient specific estimation of leaflet strain from 3D images of heart valves

**Published:** 2025-10-08

**Authors:** Wensi Wu, Matthew Daemer, Jeffrey A. Weiss, Alison M. Pouch, Matthew A. Jolley

**Affiliations:** 1 Department of Mechanical Engineering and Applied Mechanics, University of Pennsylvania, Philadelphia, PA, USA.; 2 Cardiovascular Institute, Children’s Hospital of Philadelphia, Philadelphia, PA, USA.; 3 Department of Anesthesiology and Critical Care Medicine, Children’s Hospital of Philadelphia, Philadelphia, PA, USA.; 4 Department of Biomedical Engineering, University of Utah, Salt Lake City, UT, USA.; 5 Scientific Computing Institute, University of Utah, Salt Lake City, UT, USA.; 6 Department of Radiology and Bioengineering, University of Pennsylvania, Philadelphia, PA, USA.

**Keywords:** feature tracking, leaflet strain, cardiac valves, valvular heart disease

## Abstract

Valvular heart disease is prevalent and a major contributor to heart failure. Valve leaflet strain is a promising metric for evaluating the mechanics underlying the initiation and progression of valvular pathology. However, robust and generalizable methods for noninvasively quantifying valvular strain from clinically acquired patient images remain limited. In this work, we present a novel feature-tracking framework for quantifying leaflet strain in atrioventricular valves using 3D echocardiographic images of pediatric and adult patients. Our method demonstrated superior accuracy in the assessment of anatomical deformation and strain of heart valves compared to other point-based approaches, as verified against a finite element benchmark. Further, our approach can robustly track inter-phase deformation of valves across highly variable morphologies without parameter tuning. Our analysis revealed that a median and interquartile range of the 1^st^ principal strain greater than 0.5 is associated with leaflet billow (prolapse). Further investigation of the biomechanical signatures of heart valve disease has the potential to enhance prognostic assessment and longitudinal evaluation of valvular disease.

## 1 Introduction

Valvular heart disease (*e.g.*, valve regurgitation or stenosis) is a prevalent cardiac condition that contributes substantially to the development of heart failure across all age groups [1–5]. Echocardiographic imaging is the clinical gold standard for evaluating morphological abnormalities of the heart valves (*e.g.*, leaflet billowing, prolapse, and tenting) [6]. 3D echocardiography (3DE) in particular enables 3D visualization of dynamic valve geometry, as well as quantification of functional metrics such as regurgitant volume and effective orifice area, which are critical indicators of disease severity [7–9]. However, global morphological features alone are not sufficient to characterize the biomechanical environment that governs disease initiation, progression, or the long-term durability of surgical repair. Strain analysis, in contrast, provides a detailed map of local tissue deformation that reflects tissue stiffness. This information may serve as a proxy for identifying leaflet regions at increased risk of degeneration [10–13]. Reliable non-invasive assessment of leaflet strain from 3D images thus holds great potential for advancing prognostic evaluation of valvular disease and improving predictions of surgical repair durability [14, 15].

Implications of leaflet strain on valve health and disease have been extensively investigated using finite element analysis (FEA) [16–21]. While these seminal studies have provided important insights into the fundamental mechanisms of leaflet biomechanics, the fidelity of FEA is highly dependent on how accurately the model reproduces the native valvular system. To date, patient-specific FEA of heart valves remains limited by the spatial resolution of 3DE imaging and requires simplification and individualized adjustment of models, especially for chordal structures [16, 21]. Further, many of these studies also depend on leaflet tissue properties derived from animal or cadaver experiments, which further confounds simulation outcomes. The lack of a generalized framework for patient-specific valve modeling leads to user bias and variability, which may limit the reliability of these models in clinical practice.

Feature tracking techniques, including image intensity–based registration [22–24] and point–based registration [25, 26], present an alternative approach for estimating leaflet strain. Feature tracking approximates local tissue deformation by aligning geometric features between image frames. This characteristic eliminates the need for prior knowledge of material properties or chordal structure, making this approach better suited for clinical translation. Notably, myocardial strain analysis has been widely adopted clinically to assess ventricular function. Multiple population studies suggested that myocardial strain analysis provides added value in improving diagnostic accuracy and prognostication in heart failure [27–31]. However, the application of registration-based approaches for valvular strain analysis remains in its infancy, with only a few notable studies to date [14, 15, 32]. This is primarily attributed to the challenge posed by large and rapid geometric changes in the leaflets during the transitions between open and closed time frames. While registration-based approaches hold considerable promise for valvular strain analysis, existing studies have focused exclusively on mitral valves (MVs), with most algorithms being proprietary or limited by reliance on commerical software designed for MVs alone [14, 15, 32].

To address these limitations, we sought to develop a robust open-source, registration-based strain quantification framework for the assessment of diverse valve morphologies, including patients with congenital heart disease. Our method incorporates a novel distance-decay function into the Gaussian prior of the coherent point drift algorithm to robustly track geometries with substantial deformation [26, 33]. The proposed method was verified using an FEA benchmark example, where it achieved more accurate shape and strain estimation than other published point-based registration techniques. To further demonstrate robustness across a broad range of valve morphologies and types, we applied this verified framework to three patient cohorts, including adult and pediatric patients with congenital heart disease. Reliable feature tracking and strain quantification were achieved in both MVs and tricuspid valves (TVs) in patients across a broad age range, from infants to older adults with both normal and diseased morphologies.

## 2 Materials and Methods

**Fig. 1:**
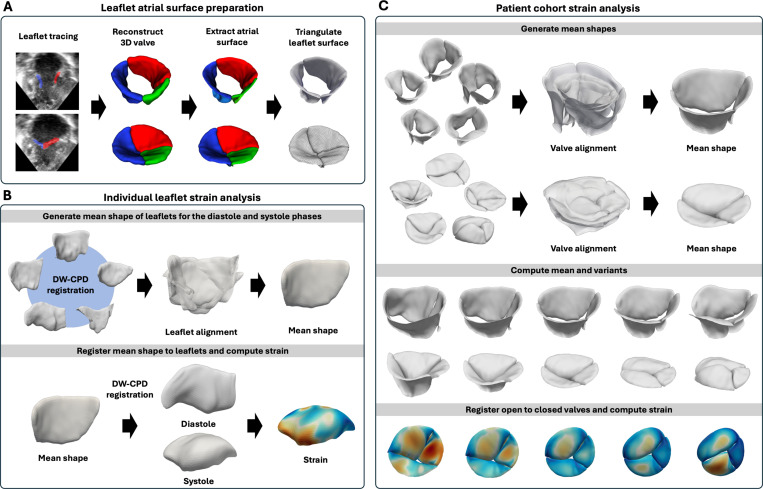
Registration-informed strain quantification framework. (**A**) The atrial surfaces of the valves were extracted from segmentation models and triangulated with 1000 points uniformly distributed across each leaflet. (**B**) Within each patient cohort, one diastolic valve was randomly selected as the reference. Leaflets of the same anatomical category were registered to the corresponding reference leaflet to establish point-to-point correspondence. The registered leaflets were then aligned, and the mean shape was estimated using generalized procrustes analysis. The mean shape was registered to both the diastolic and systolic frames of that leaflet and leaflet deformation was derived to compute strain on individual leaflets. (**C**) For patient cohort analysis, mean valve shapes at diastole and systole were first estimated using generalized procrustes analysis. Principal component analysis was applied to determine shape variation within each cohort. Finally, valves were registered between the open and closed phases to estimate systolic valve strain.

Our proposed registration-informed strain quantification framework is presented in Fig. 1. In all subjects, the areal and 1^st^ principal strain at the mid-systolic frame were computed using the mid-diastolic frame as reference. The code of our strain calculation framework will be made available at https://github.com/ww382/registration_informed_valve_strain.

### 2.1 Three-dimensional echocardiographic image processing

#### 2.1.1 Atrioventricular valve reconstruction

3DE images of pediatric TVs from patients with HLHS (n = 11; 2 TEE and 9 TTE) and MVs (n = 10; 3 TEE and 7 TTE) were obtained from an existing database at the Children’s Hospital of Philadelphia. These images were acquired on a Philips Epiq system (Philips Medical, Boston, MA). In addition, transesophageal 3DE images from adult patients (n = 10) with normal MV morphology were obtained from an existing database at the University of Pennsylvania and were acquired on a Philips iE33 system (Philips Medical Systems, Andover, MA). The pediatric data were imported into the SlicerHeart module [9, 34, 35] in 3D Slicer for segmentation, while the adult data were manually traced using ITK-SNAP [36]. The valve leaflets at the mid-diastolic and mid-systolic frames of the cardiac cycle were segmented by an expert observer to facilitate the 3DE-informed strain analysis. These were multi-label segmentations with a separate label for each leaflet. Image voxels corresponding to the leaflets were manually labeled and converted into 3D models (Fig. 1A), as described in [7, 8, 37, 38]. The collection and analysis of these data were approved by the Institutional Review Boards at the Children’s Hospital of Philadelphia and the University of Pennsylvania.

#### 2.1.2 Atrial surface mesh creation

The atrial surface of each leaflet segmentation was extracted using the Leaflet Analysis tool in SlicerHeart. In particular, a closed curve was defined along the margin of the leaflet that separates the atrial and ventricular sides of the segmentation model. The model was then clipped along this curve to isolate the atrial surface. A custom Python script that utilized the PyACVD library [39] was subsequently used to process the extracted surface. First, the extracted surface was cleaned by removing isolated and unreferenced points and then smoothed using Taubin smoothing (10 iterations, pass band = 0.05). Second, the surface was remeshed with 1,000 nodes uniformly distributed across the atrial surface.

### 2.2 Distance-weighted coherent point drift (CPD) registration

The development of our DW-CPD registration approach was inspired by the original CPD formulation for nonrigid point set registration [26]. In the standard CPD framework, the fixed point set is represented as the centroids of a Gaussian mixture model (GMM) with isotropic covariances and uniform priors. The moving point set is transformed to the fixed point set through iterative expectation–maximization (EM) optimization to maximize the posterior correspondence probabilities (posterior probability function). The deformation field is regularized by the motion coherence theory [33], which penalizes non-smooth variations in the velocity field. This ensures that neighboring points maintain coherent movement and preserve the topological structure of the point set.

While effective in many applications, the original CPD formulation can lead to unphysical global distortions in heart valve modeling, particularly when substantial changes in leaflet concavity between the open and closed configurations (*e.g.*, in the case of a billowing leaflet). To improve the generalizability of CPD across diverse valve geometries, we introduce an adaptive, distance-weighted modification to the mixture model priors. In the new formulation, the prior probability of correspondence is increased for spatially proximate point pairs across the moving and target sets, while the likelihood of correspondence is weakened for more distant pairs. The motivation of this strategy reduces spurious global matches, mitigates large-scale geometric distortions, and thus yields more accurate leaflet tracking.

#### 2.2.1 Gaussian mixture models

Consider two point clouds $X={\left(x_{1},\ldots ,x_{N}\right)}^{T}\in {\mathbb{R}}^{N\times 3}$ and $Y={\left(y_{1},\ldots ,y_{M}\right)}^{T}\in {\mathbb{R}}^{M\times 3}$, where X denotes the nodal coordinates of the mid-systolic mesh (fixed frame) with N nodes and Y denotes the nodal coordinates of the mid-diastolic mesh (moving frame) with M nodes within each valve. In standard CPD, points in Y were considered GMM centroids with uniform priors and equal, isotropic covariances. The resulting mixture density for a point x∈X is

(1) $$p(x)=\frac{1}{M}\sum_{m=1}^{M}\frac{1}{{\left(2\pi \sigma^{2}\right)}^{D/2}}\exp\left(-\frac{{\left\|x-y_{m}\right\|}^{2}}{2\sigma^{2}}\right),$$

with D=3 and σ denotes the standard deviation of the Gaussian distribution. In this present work, all mid-systolic and mid-diastolic meshes have the same number of nodes. To account for uncertainty in point correspondence (e.g., noisy points or outliers), CPD introduces an additional uniform component with weight w∈[0,1), yielding

(2) $$p(x)=w\frac{1}{N}+(1-w)\frac{1}{M}\sum_{m=1}^{M}\frac{1}{{\left(2\pi \sigma^{2}\right)}^{D/2}}\exp\left(-\frac{{\left\|x-y_{m}\right\|}^{2}}{2\sigma^{2}}\right).$$

The transformation T that maps the GMM centroids $y_{m}$ to their deformed locations is estimated by minimizing the regularized negative log-likelihood function

(3) $$E\left(T,\sigma^{2}\right)=-\sum_{n=1}^{N}\log p\left(x_{n}\right)+\frac{\alpha }{2}\phi (T),$$

where $p\left(x_{n}\right)$ is the mixture model likelihood in Eq. 2, α is a weighting constant, and ϕ(T) is a regularization function that enforces motion coherence and smoothness in the displacement field. Herein, T is denoted as

(4) $$T(Y,v)=Y_{0}+v(Y),$$

where $Y_{0}$ is the initial nodal positions in the moving point set and v is the displacement function.

#### 2.2.2 Expectation-maximization optimization

CPD employs the EM algorithm to iteratively refine the nodal correspondences and registration parameters, as direct optimization of the log-likelihood is intractable. In the expectation (E) step, the posterior probability of each data point $x_{n}\in X$ is given by

(5) $$P_{mn}=\frac{\exp\left(-\frac{{\left\|x_{n}-T\left(y_{m}\right)\right\|}^{2}}{2\sigma^{2}}\right)}{\sum_{k=1}^{M}\exp\left(-\frac{{\left\|x_{n}-T\left(y_{k}\right)\right\|}^{2}}{2\sigma^{2}}\right)+{\left(2\pi \sigma^{2}\right)}^{D/2}\frac{w}{1-w}\frac{M}{N}},$$

where P is the matrix containing the probability of correspondence. In our DW-CPD approach, we modify this expression to prioritize point correspondences in neighboring points by introducing a distance decay function defined as

(6) $$P_{mn}\propto P_{mn}\cdot \exp\left(-\gamma \left\|x_{n}-T\left(y_{m}\right)\right\|\right),$$

where γ is a distance decay factor, with a larger γ, the stronger the penalty is for enforcing the nodes in the moving frames to align very closely with the nodes in the fixed frame. Depending on the application, one may adjust γ to impose tighter (ensure node proximity) or looser (enables positional flexibility) locality constraints between the moving and fixed frame.

In the maximization (M) step, we solve for the matrix of the Gaussian kernel weights, $W\in {\mathbb{R}}^{M\times D}$, using the posterior probability function, P, in the E step

(7) $$\left(G+\alpha \sigma^{2}\mathrm{diag}{\left(P1\right)}^{-1}\right)W=\mathrm{diag}{\left(P1\right)}^{-1}PX-Y,$$

where 1 is a column vector of ones of length N and $G\in {\mathbb{R}}^{M\times M}$ is a symmetric Gram matrix with elements $g_{ij}=\exp\left(-\frac{1}{2}{\left\|\frac{y_{0i}-y_{0j}}{\beta }\right\|}^{2}\right)$, where β defines the width of the Gaussian filter. In the present work, we set α=1, β=5, and γ=0.05.

### 2.3 3DE-informed strain analysis in vivo

Here, we present our workflow for strain analysis on individual leaflets (Fig. 1B) as well as population-based analysis (Fig. 1C) based on nodal correspondences obtained from DW-CPD.

#### 2.3.1 Individual lealfet analysis

Leaflet geometry in atrioventricular valves is highly heterogeneous, particularly in congenital heart disease [37]. To ensure robust registration, we first used the VTK Python module to align and rotate individual leaflets of the same label (e.g., anterior, posterior, septal) by their centroids so that their atrial surfaces face the same orientation. We manually checked the aligned leaflets to make sure the annular and free edges across all leaflets were in the same direction. A representative leaflet of diastole from one randomly selected patient was then chosen as the reference, and all other leaflets of the same anatomical category were subsequently registered to it using DW-CPD to establish intra-subject correspondences. From the registered leaflets, a mean shape was generated using generalized procrustes analysis. The mid-diastolic and mid-systolic point sets were then registered to this mean shape to establish point correspondences between the two phases. Finally, the deformation gradient and strain were computed from the nodal coordinates of the mid-diastolic and mid-systolic point sets.

Both areal strain and the 1^st^ principal Green-Lagrange strain were considered in the present work. The areal strain of the mesh is formulated as

(8) $$E_{\text{areal}}=\frac{A_{\text{def}}-A_{\text{ref}}}{A_{\text{ref}}},$$

where $A_{\text{def}}$ and $A_{\text{ref}}$ contain the area of each cell in its reference (mid-diastolic) and deformed (mid-systolic) state, respectively. The Green-Lagrange strain is formulated as

(9) $$E_{\text{Green-Lagrange}}=\frac{1}{2}(C-I),$$

where C is the right Cauchy-Green tensor and I is the identify matrix. C is formulated as $C=F^{T}F$ where F, the deformation gradient tensor, is computed as $F=\left[t_{1};t_{2};n_{\text{ref}}\right].{\left[T_{1};T_{2};n_{\text{def}}\right]}^{-1}$. Herein, $t_{1}$ and $t_{2}$ are the tangent vectors of each triangular cell in the reference state. The normal vector is computed as $n_{\text{ref}}=\frac{t_{1}\times t_{2}}{\left\|t_{1}\times t_{2}\right\|}$. $T_{1}$ and $T_{2}$ are the tangent vectors in the deformed state.

#### 2.3.2 Population-based whole valve analysis

With one-to-one correspondence established across all leaflets, we applied generalized Procrustes analysis to iteratively align the valves and compute a mean shape for each patient cohort. Because of substantial variability in valve geometries, particularly within the HLHS TV cohort, some valves occasionally had misoriented leaflets, which could result in erroneous mean shape calculations. In such cases, the valves were manually rotated to the correct orientation. Principal component analysis was then performed to quantify shape variations at the mid-diastolic and mid-systolic frames. DW-CPD registration was performed on each pair of diastole-systole valve shapes, and strain was subsequently computed.

## 3 Results

We performed comprehensive leaflet strain analyses on three patient cohorts from the Children’s Hospital of Philadelphia and the University of Pennsylvania. Detailed verification results on shape agreement using our proposed method, together with a comparative analysis against other point-based registration techniques, are provided in Section 3.1. Section 3.2 presents strain analysis of TVs in patients with hypoplastic left heart syndrome (HLHS). Section 3.3 provides strain analysis of pediatric MVs, including both normal and a range of pathologic conditions, such as rheumatic mitral regurgitant valve and Marfan syndrome. Section 3.4 covers the strain analysis of normal adult MVs. In this context, “normal” indicates valves that coapt properly without regurgitation or tissue calcification. However, these adult patients had other cardiac conditions, such as aortic stenosis or coronary artery disease, at the time of imaging. All registration-based strain analyses were performed on a MacBook Pro with an Apple M2 Max chip (Apple Computer, Cupertino, CA). The average computational time for registration and strain estimation combined was 14.2 seconds per valve (with 3000 nodes per valve).

### 3.1 Verification results

An image-derived mitral valve (MV) model from Wu et al.[40] was used as the benchmark to verify the proposed algorithm. The ground truth valve deformation, areal strain, and 1^st^ principal Green Lagrange strain were generated in FEBio using the same model setup described in the original study. The strain formulation is described in Section 2.3. We compared the registration and strain estimation performance of our method against established point-based registration approaches: Bayesian-CPD (BCPD) [41], support vector registration (SVR) [42], and traditional CPD [26]. Probreg [43] was used to perform the BCPD and SVR registration.

**Fig. 2:**
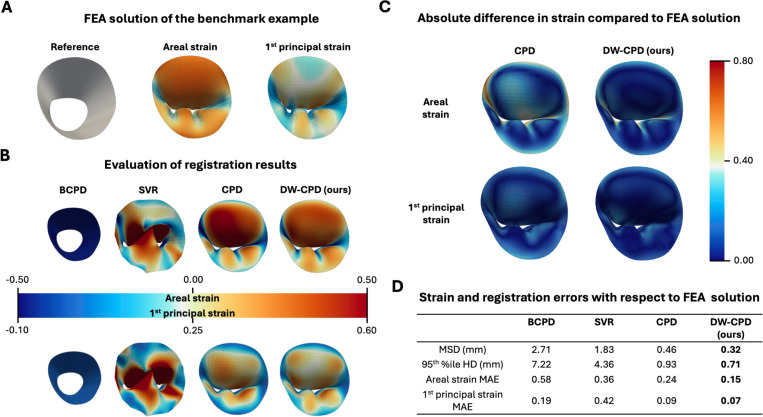
Registration verification results. (**A**) Ground truth areal and 1^st^ principal strain of an image-derived MV finite element model were used to compare the performance of the proposed and established feature-tracking methods. (**B**) Qualitative comparisons of the deformed geometry and strain distributions are provided. Both CPD and DW-CPD successfully captured the large valve deformation and the strain patterns. (**C**) Both CPD and DW-CPD tend to overpredict strain in regions with high curvature, with larger errors observed in CPD. (**D**) Quantitative evaluation of registration and strain estimation errors is summarized. The proposed DW-CPD consistently achieved the lowest shape discrepancy and strain error across all metrics.

The reference areal and 1^st^ principal strain fields are shown in Fig. 2A. Fig. 2B provides a qualitative comparison between the estimated and ground truth strain maps. As illustrated, both BCPD and SVR failed to register the deformed geometry of the MV. In contrast, the standard CPD and our proposed DW-CPD successfully captured both the deformed shape and the associated strain patterns. The absolute difference between registration-informed strain and the FEA solution from CPD and DW-CPD approach is plotted in Fig. 2C. We observed higher absolute errors in both areal and first principal strain in regions with high curvature and bending (e.g., near the commissure folds) for both methods. CPD overpredicted strain more than DW-CPD, with up to 0.8 absolute error in area strain. Quantitative comparisons reporting errors in shape agreement and strain estimation are summarized in Fig. 2D. Shape agreement was assessed using the mean symmetric distance (MSD) and the 95^th^ %ile Hausdorff distance (HD), while strain accuracy was evaluated using the mean absolute error (MAE) of the areal and 1^st^ principal strains. Across all four metrics, our method consistently outperformed others, achieving an MSD of 0.32 mm, a 95^th^ %ile HD of 0.71 mm, an areal strain MAE of 0.15, and a 1^st^ principal strain MAE of 0.07.

**Fig. 3:**
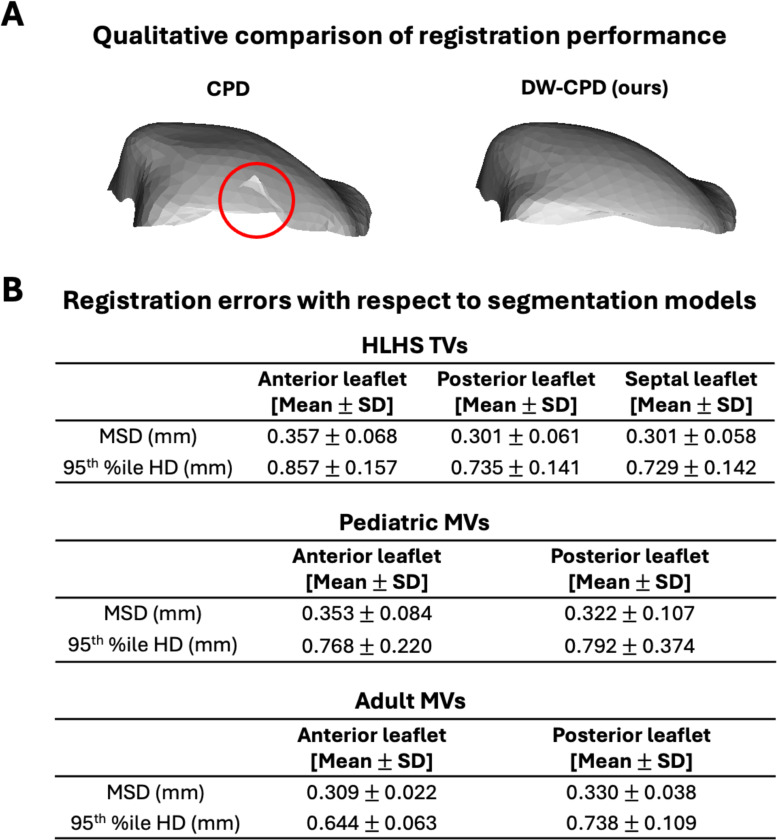
Registration performance. (**A**) This example illustrates that CPD resulted in self-penetration at the leaflet edge, while the proposed DW-CPD eliminated this defect with improved registration fidelity. (**B**) DW-CPD was applied to 3 groups of atrioventricular valves and verified against ground truth segmentation. Strong shape agreement was observed, with MSD values below 0.4 mm and 95^th^ %ile HD values below 1 mm across all leaflets.

We applied the proposed DW-CPD feature-tracking method to estimate leaflet deformation in three patient cohorts: (1) TV in patients with HLHS, (2) pediatric MVs, and (3) adult MVs. Fig. 3A shows an example where the CPD method produced a registration artifact at the leaflet edge, causing it to fold inward and protrude, resulting in self-penetration. In contrast, DW-CPD accurately tracked the leaflet deformation, eliminating this defect. The mean and SD of the MSD and 95^th^ percentile HD for each leaflet within each cohort are summarized in Fig. 3B. DW-CPD was used to inform the strain calculation among the patient cohorts. Overall, the registered leaflets achieved strong agreement compared with the ground truth segmentation models. Among the patient groups, the anterior leaflet of HLHS TVs showed the largest errors, with an average MSD of 0.357mm ± 0.068 and a 95^th^ %ile HD of 0.857 mm ± 0.157.

### 3.2 Strain assessment of pathological tricuspid valves

#### 3.2.1 Patient population

**Fig. 4:**
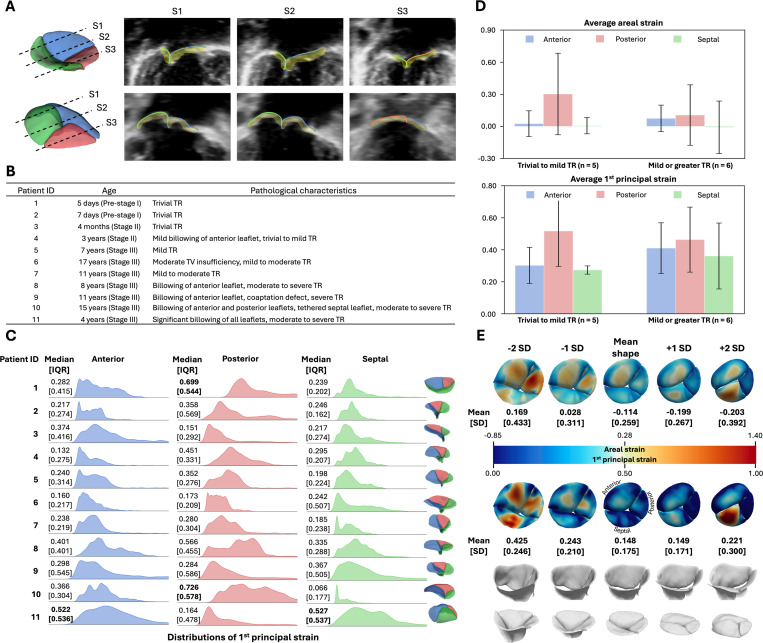
Pediatric tricuspid valve analysis. (**A**) The estimated systolic atrial surfaces were superimposed onto 3D TEE images along with the ground truth segmentation models. Excellent registration accuracy was obtained, as demonstrated in the visualization of the three cut planes shown. Quantitative error metrics were reported in the Supplemental text. (**B**) The study cohorts included patients spanning a wide age range, from 5 days to 17 years. For each patient, the surgical stage at the time of image acquisition, as well as the pathological characteristics of the TVs, were reported. (**C**) The distributions of 1^st^ principal strain, as well as their median and IQRs, were compared across the three leaflets in all 11 patients. Billowing leaflets were typically characterized by a high median and strain IQR, with a relatively uniform strain distribution profile. (**D**) Average areal strain and 1^st^ principal strain were compared between the trivial to mild and mild or greater TR cohorts. The trivial to mild cohort exhibited lower areal and 1^st^ principal strain in the anterior leaflet but higher values in the posterior leaflet relative to the mild or greater cohort. Both cohorts showed near zero areal strain in the septal leaflet, while the mild or greater cohort demonstrated higher 1^st^ principal strain in the septal leaflet. (**E**) Population-based strain analysis was performed. 1^st^ principal strain and IQRs increase as the valve morphology deviates from the mean shape.

This study cohort comprises 11 pediatric patients diagnosed with HLHS: 2 patients with HLHS prior to stage I palliation, 2 with stage II HLHS, and 7 with stage III HLHS following Fontan palliation. The patient cohort was randomly selected from our database. Patients’ age at the time of image acquisition ranges from 4 months to 17 years. Of the 11 patients, 5 had trivial to mild tricuspid regurgitation (TR), and 6 developed mild or greater TR. Examples of the tracked leaflet atrial surface, along with segmentation ground truth, overlaid on 3DE images, are shown in Fig. 4A. Descriptions of the TV pathological characteristics (drawn from clinical reports) and the age associated with each patient are summarized in Fig. 4B.

#### 3.2.2 Strain characteristics on individual leaflet

The areal and 1^st^ principal strains at the mid-systole frame were calculated for each TV. Fig. 4C shows ridgeline plots of the 1^st^ principal strain distributions for each leaflet in each patient, along with the corresponding median and interquartile range (IQR). Qualitatively, billowing leaflets showed a broader and flatter strain distribution, while valves with trivial to mild TR exhibited more sharply defined peaks and a narrower spread. This pattern indicates that strain in valves with trivial to mild TR is more localized, while strain in billowing leaflets is more uniformly distributed. Additionally, we observed that valves with a higher regurgitant grade tend to be accompanied by higher median and IQR of strain. The leaflets with both mean and IQR values greater exceed 0.5 are highlighted in bold. Of the 4 leaflets identified, 3 were confirmed as billowing.

We compared the average areal and 1^st^ principal strain in the trivial to mild TR and mild or greater TR cohorts. The mean and standard deviation (SD) of the average areal strain and 1^st^ principal strain on each leaflet are shown in Fig. 4D. The average areal strain quantifies the relative change in leaflet surface area between diastolic and systolic frames, whereas the average 1^st^ principal strain represents the mean maximum tensile strain across the leaflet. With respect to areal strain, both groups exhibited negligible changes in septal leaflet area while demonstrating increased anterior and posterior surface area. Both the trivial to mild TR and mild to greater TR cohorts showed similar anterior areal strain, although the trivial to mild TR had slightly lower values (0.025 ± 0.120 vs. 0.075 ± 0.124). However, posterior areal strain was substantially higher in the trivial to mild TR cohort compared to the mild or greater cohort (0.302 ± 0.381 vs. 0.106 ± 0.282). This suggests that patients in the trivial and mild cohort have more extensible posterior leaflets. Consistent with the areal strain findings, the trivial to mild TR cohort exhibited lower average 1^st^ principal strain in the anterior leaflet compared to the mild or greater cohort (0.303 ± 0.113 vs. 0.410 ± 0.158), but higher average 1^st^ principal strain in the posterior leaflet (0.517 ± 0.221 vs. 0.463 ± 0.203). For the septal leaflet, the average 1^st^ principal strain was 0.273 ± 0.026 in the trivial to mild cohort and 0.361 ± 0.206 in the mild or greater cohort.

#### 3.2.3 Population study of strain patterns across the patient cohort

Principal component analyses (PCA) of the TVs at mid-diastole and mid-systole were performed independently to determine the mean valve shape and its standard deviations (SD) across the 11 patients. Leaflet strains were computed by tracking point-to-point correspondences between the open and closed valve geometries using our distance-weighted coherent point drift (DW-CPD) registration approach. The resulting spectrum of valve morphologies and associated strain information is shown in Fig. 4E. Within this spectrum, the valve appeared most tethered at −2 SD from the mean shape and most billowing at +2 SD. Both ends of the morphological spectrum demonstrated markedly elevated strains relative to the mean shape. In the most tethered valve, high strain was distributed across all three leaflets, whereas in the most billowing valve, high strain was concentrated on the septal leaflet.

The mean shape exhibited relatively low strains (–0.114 ± 0.259 average areal strain and 0.148 ± 0.175 average 1^st^ principal strain). In contrast, the most tethered valve showed an average areal strain of 0.169 ± 0.433 and a 1^st^ principal strain of 0.425 ± 0.246, while the most billowing valve demonstrated an average areal strain of −0.203 ± 0.392 and a 1^st^ principal strain of 0.221 ± 0.300. For both areal and 1^st^ principal strain, the SD of the strain distribution increased as valve geometries deviated further from the mean shape. These findings suggest that both elevated 1^st^ principal strain and greater strain variability, represented by SD, are associated with pathological TV morphology.

### 3.3 Strain assessment of normal and diseased pediatric mitral valves

#### 3.3.1 Patient population

**Fig. 5:**
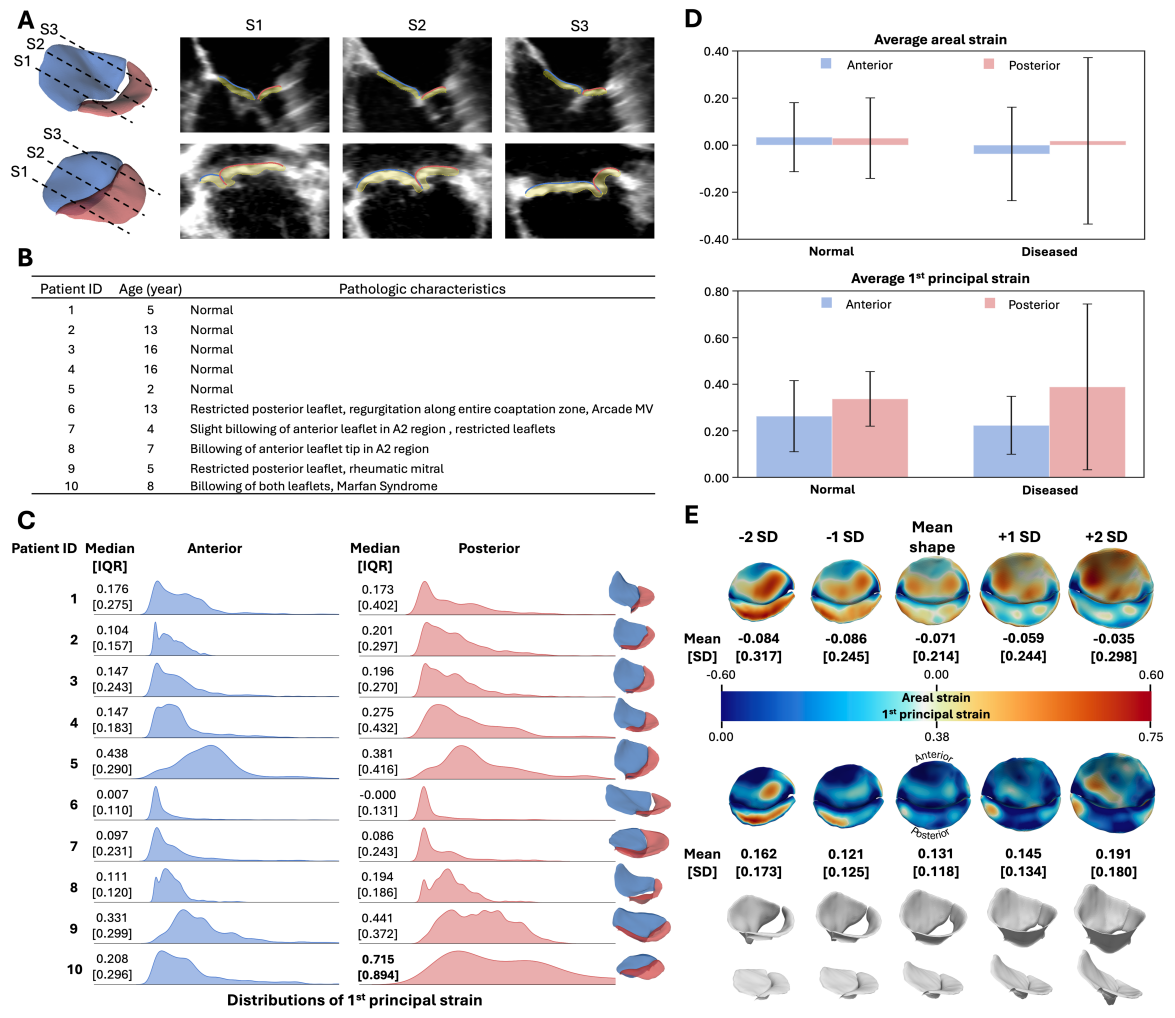
Pediatric mitral valve anlaysis. (**A**) Visualization of the ground truth segmentation models and estimated atrial surfaces were superimposed onto 3D TEE images on the three cut planes shown. Quantitative error metrics were reported in the Supplemental text. (**B**) The study cohorts included patients from 2 to 16 years old at the time of image acquisition. The MV pathological characteristics for each patient were provided. (**C**) The median, IQR, and distribution of 1^st^ principal strain were compared across the anterior and posterior leaflets in all 10 patients. Patient 10 was the only case with leaflet strain exhibiting a median and IQR greater than 0.5, which also corresponded to billowing morphology. (**D**) Average areal strain and 1^st^ principal strain were compared between the normal and diseased cohorts. Both cohorts showed negligible changes in leaflet surface area and demonstrated higher average 1^st^ principal strain in the posterior leaflet than in the anterior leaflet. (**E**) Population-based strain analysis showed that higher average 1^st^ principal strains at both ends of the shape spectrum compared with the mean shape. In addition, strain SD increased as valve morphology deviated from the mean.

This study cohort included 10 pediatric patients, aged 2 to 16 years at the time of image acquisition. Of these, 5 had normal coaptation, 3 demonstrated billowing of one or more leaflets, and 2 had restricted posterior leaflets. Further, the cohort included 1 patient with an MV arcade, 1 with rheumatic MV, and 1 with Marfan syndrome. Fig. 5A shows the superimposition of ground truth segmentation and the estimated atrial leaflet surface onto 3D TEE images from two representative patients. The ages and clinical diagnoses of all patients are summarized in Fig. 5B.

#### 3.3.2 Strain characteristics on individual leaflet

Ridgeline plots comparing the distributions, median and IQRs of 1^st^ principal strain for the anterior and posterior leaflets are shown in Fig. 5C. The diagnoses among MV patients in this cohort were highly heterogeneous, resulting in considerable variability in strain distribution patterns. We highlighted the posterior leaflet of Patient 10, which showed a median strain and IQR greater than 0.5. Notably, the clinical note described this leaflet as billowing. This finding further suggests that the median and IQR of 1^st^ principal strain could serve as a promising biomechanical signature for diagnosing pathological leaflets.

We organized the patient cohort into normal and diseased groups and compared the average areal and 1^st^ principal strains (Fig. 5D). Neither group exhibited significant changes in leaflet area between the mid-diastolic and mid-systolic frames. In the normal cohort, the average areal strain was 0.034 ± 0.147 in the anterior leaflet and 0.029 ± 0.171 in the posterior leaflet. In the diseased cohort, the corresponding values were −0.038 ± 0.198 and 0.018 ± 0.354.

The posterior leaflets exhibited higher 1^st^ principal strain than the anterior leaflets in both normal and diseased cohorts. In the normal cohort, the average 1^st^ principal strain was 0.262 ± 0.152 in the anterior leaflet and 0.337 ± 0.117 in the posterior leaflet. In the diseased cohort, the corresponding values were 0.223 ± 0.124 and 0.388 ± 0.356. No clear differences in 1^st^ principal strain were observed between the two cohorts, as the inter-cohort differences for each leaflet were less than 0.05.

#### 3.3.3 Population study of strain patterns across the patient cohort

The variations of MV morphology in this cohort and the associated strain patterns are shown in Fig. 5E. Within the spectrum, the MV with the smallest anterior–posterior annular diameter corresponded to −2 SD from the mean shape, whereas the largest corresponded to +2 SD. The MVs at both ends of the spectrum exhibited higher peak strains than the mean shape. At −2 SD, the MV showed uniformly elevated strain across the entire posterior leaflet.

Although differences in MV strain were modest, we observed a unidirectional increase in mean areal strain from −0.084 to −0.035 as MV morphology shifted from −2 to +2 SD of valve shapes. In contrast, both ends of the morphological spectrum exhibited higher average 1^st^ principal strain than the mean shape: 0.162 at −2 SD and 0.191 at +2 SD, compared to 0.131 in the mean shape. Similar to the TV analysis, the SD of areal and 1^st^ principal strain increased as valve geometries deviated further from the mean.

### 3.4 Strain assessment of adult mitral valves

#### 3.4.1 Patient population

**Fig. 6:**
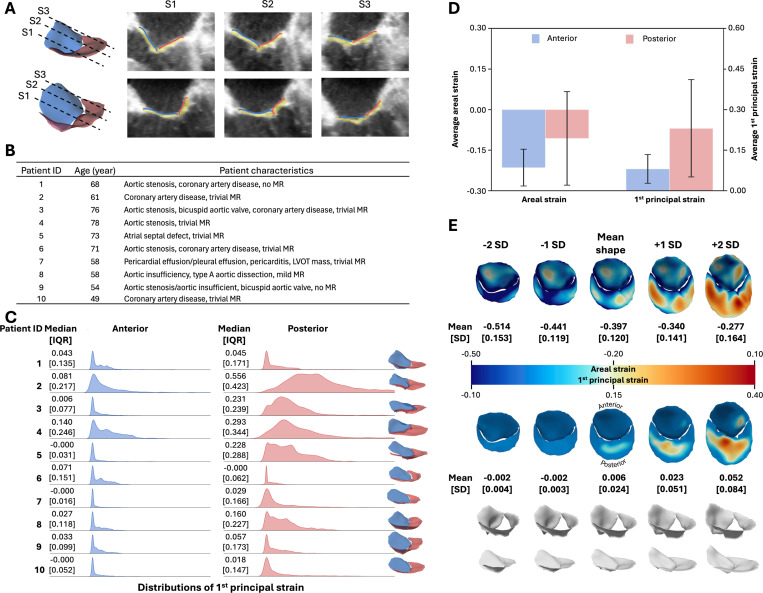
Adult mitral valve anlaysis. (**A**) Visualization of the ground truth segmentation models and estimated atrial surfaces were superimposed onto 3D TEE images. Quantitative error metrics were reported in the Supplemental text. (**B**) The study included adult patients aged 49 to 78 years at the time of image acquisition. All patients had normal MV function but presented with other cardiac conditions. (**C**) Narrow 1^st^ principal strain distributions were observed in all MV leaflets. Further, the median and IQR of the strain distributions were below 0.5 across all 10 patients. (**D**) Both anterior and posterior leaflets exhibited negative areal strain, which may suggest substantial annular contraction between the mid-diastolic and mid-systolic frames in this cohort. For 1^st^ principal strain, consistent with the pediatric MV study, the posterior leaflet showed higher strain than the anterior leaflet. (**E**) In the population-based strain analysis, average areal strain and 1^st^ principal strain both increased with increasing anterior–posterior diameter.

This study cohort comprised 10 adult cardiac surgery patients, aged 49 to 78 years at the time of intra-operative image acquisition. All patients demonstrated normal MV function with no to mild mitral regurgitation (MR), though each had non-mitral cardiac pathology. The ground truth segmentation and the estimated atrial leaflet surface overlaid onto 3D TEE images from two representative patients are presented in Fig.6A. Further, Fig.6B summarizes the ages and clinical diagnoses of the entire cohort. Unlike the previous two cohorts, none of the patients in this group were identified with billowing leaflets.

#### 3.4.2 Strain characteristics on individual leaflet

Fig. 6C presents ridgeline plots comparing the 1^st^ principal strain in the anterior and posterior leaflets. In most patients, the MV leaflets exhibited narrow strain distributions with low median and IQR values. An exception was Patient 2, whose posterior leaflet demonstrated a broader distribution with a median of 0.556 and an IQR of 0.423.

Fig. 6D compares the average areal and 1^st^ principal strain of each leaflet across the patient cohort. Interestingly, negative average areal strain was observed in both leaflets (anterior: −0.215 ± 0.068; posterior: −0.107 ± 0.173). This may indicate a significant contraction of the annulus within the mitral valve in the adult population. However, further analysis is warranted to identify the causes of the negative average areal strain. For 1^st^ principal strain, the posterior leaflet exhibited higher values than the anterior leaflet (0.231 ± 0.180 vs. 0.081 ± 0.053).

#### 3.4.3 Population study of strain patterns across the patient cohort

Fig. 6E illustrates the spectrum of MV morphologies in this cohort along with their associated strain distributions. Consistent with the pediatric cohort, the valve with the shortest anterior–posterior annular diameter corresponded to −2 SD from the mean shape, whereas the longest corresponded to +2 SD. In this cohort, both average areal strain and 1^st^ principal strain increased as the anterior–posterior annular diameter increased from −2 SD to +2 SD of valve shapes. In terms of strain heterogeneity, the SD of areal strain increased as the valve geometries deviated further from the mean shape. Conversely, the SD of the 1^st^ principal strain increased undirectionally from −2 SD to +2 SD of the valve shape.

## 4 Discussion

### 4.1 General comments

In this study, we present an open-source, registration-based framework for quantifying leaflet strain in atrioventricular valves. We verified our implementations against FEA benchmark, and excellent shape and strain agreements were achieved. Compared with BCPD [41], SVR [42], and traditional CPD [26], our method consistently achieved superior performance across all metrics. To assess robustness to morphological variation, we applied the framework to three patient cohorts: (1) TVs in patients with HLHS, (2) pediatric MVs, and (3) adult MVs. The dataset spans normal to regurgitant valves and ages from neonates to the late seventies. This constitutes the first human analysis of tricuspid valve strain quantification comparing healthy and diseased cohorts, and the most heterogeneous patient population studied to date. Despite substantial variation in valve geometry across patients and cohorts, the same registration parameters produced accurate results in all cases. This highlights the generalizability of the framework. Across cohorts, the median and IQR of the 1^st^ principal strain distributions emerged as the most sensitive biomechanical marker of leaflet health, with elevated median and IQR values of 0.5 consistently indicating billowing.

The assessment of the biomechanical strain of valves is central to the understanding of the mechanical environment and resulting tissue and cellular changes underlying disease initiation and progression [10–13]. FEA has been widely used to examine the effects of annular dynamics [19, 20], papillary muscle displacement [44], constitutive properties [45, 46], and annular geometry [47] on valve function. Yet most studies rely on animal or in vitro models with parameters derived from non-human tissue. Although image-derived FEA now allows patient-specific reconstructions from clinical imaging [16, 21, 48], challenges remain. Fitting of FEM based structures to image derived valve leaflet models can be challenging due to complex leaflet morphology. In addition, such approaches can be prone to user bias as a result of the need for significant local parameter tuning to reliably capture the leaflet surface with high fidelity. Further, such simulations require assumptions related to subvalvar chordal attachments or a surrogate for these forces [16, 21]. In contrast, registration-based strain offers a compelling alternative for quantifying strain from 3DE, as the formulation does not require assumptions about constitutive models and can be performed on the leaflet surfaces themselves. While registration-based approaches have been explored, application to valvular strain has been limited to mitral valve assessment with dependence on input from commercial mitral valve modeling software [15]. Further, this existing capability tracked valves from mid-systole to end-systole, which does not capture the near strain free diastolic state of an open valve as a baseline. In addition, this existing software cannot be extended to accommodate diverse valves nor allow incorporation of segmentation-based valve models, which can increasingly be rapidly generated using machine learning-based approaches [49, 50]. Finally, while 3D + time (4D) CT and cardiac magnetic resonance imaging are increasingly being utilized to evaluate valve function, there is currently no generalizable framework for analysis of valve leaflet strain across any 3D modality with sufficient temporal resolution. The need for multimodality-based assessment of valve leaflet strain across diverse valve morphologies and populations, including patients with complex congenital heart disease, is the key motivation for the present study.

To provide a summary of our strain analysis for MVs, clear differences in the characteristics of 1^st^ principal strain distributions between pediatric and adult MVs were observed. In pediatric valves, the distribution was broader with a higher IQR, whereas in adults, it was narrower and more peaked. In population-level PCA, both groups showed increasing strain IQR as valve geometries deviated from the mean shape. In adults, distinct patterns emerged in which strain in the posterior leaflet progressively increased and became more diffuse with increasing anterior–posterior diameter. In contrast, this pattern was not observed in the pediatric MV cohort. In our pediatric cohort (mean age 8.9 ± 4.9 years), the average 1^st^ principal strain was 0.262 in the anterior leaflet vs. 0.337 in the posterior leaflet in valves with normal morphology. Conversely, the average 1^st^ principal strain reported in our adult cohort (mean age 64.6 ± 9.4 years) was markely lower, with strains exhibiting 0.081 and 0.231, respectively. These findings confirm the general intuition that leaflet strain reduces with age, attributed to an increase in tissue stiffness and thickness.

### 4.2 Comparison with existing literature

Strain in TVs varies substantially across leaflets [19, 20]. In our study, patients with mild or greater regurgitation exhibited greater average areal strain and 1^st^ principal strain in the anterior leaflet, but lower values in the posterior leaflet, compared with the trivial to moderate cohort. Across both normal and diseased groups, the posterior leaflet consistently experienced the greatest stretch, followed by the anterior and septal leaflets. This pattern aligns with findings reported by Spinner et al. [44]. However, Mathur et al. identified the anterior leaflet as having the highest areal strain [19]. Analysis at the individual leaflet level further showed that billowing leaflets tend to exhibit higher IQR in the 1^st^ principal strain, suggesting greater strain heterogeneity. Strain analysis in larger cohorts is needed to verify these observations. Population-level PCA demonstrated that the normal valve, represented by the mean shape, has the lowest average strain and IQR. As valves deviate from this mean toward tethering or billowing phenotypes, both the average strain and the IQR increase.

In our MV studies, we observed higher average strain in the posterior leaflet compared with the anterior leaflet, consistent with the literature [15]. This trend was evident in our 1^st^ principal strain calculations across pediatric and adult populations, and in both normal and diseased valves. In adults, El-Tallawi et al. [15] reported average isotropic strains of 0.076 and 0.093 in the anterior and posterior leaflets, respectively, for normal MVs (mean age 55.3 ± 2.6 years). In our adult cohort (mean age 64.6 ± 9.4 years), the average areal strain in the anterior and posterior leaflets was −0.215 vs. −0.107, and the average 1^st^ principal strain was 0.081 vs. 0.231. Our results indicate that the strain measured in the posterior leaflets is approximately 2.8 times higher than that in the anterior leaflets. This value is notably higher (two times) than that reported by El-Tallawi et al. We attribute this difference to the specific time points chosen for strain calculation. El-Tallawi et al. calculated strain from mid-systole to end-systole. In contrast, our study measured strain from mid-diastole to mid-systole. Since the leaflet experiences maximum pressure near mid-systole, calculating the strain across that wider time frame (mid-diastole to mid-systole) inherently captures a larger deformation range and, consequently, results in a higher calculated strain value.

### 4.3 Limitations and area for future work

Notably, tissue thickness also influences the rate of deformation (*i.e.*, the extent to which the tissue stretches or strain rate). While our approach provided robust estimates of systolic valve deformation and strain, it did not account for spatial variation in leaflet thickness, which may influence local strain patterns. Further, factors such as signal dropouts in the echocardiographic images can introduce errors into the segmentation models. This error may subsequently degrading the accuracy of the final strain analysis. While are methods are fundamentally applicable to any 3D + time imaging modality, we only demonstrated application to echocardiography given the ready availability of images with sufficient temporal resolution. In our current analysis, we observed a promising trend suggesting that leaflet billowing is associated with an elevated median and IQR of 1^st^ principal strain, a finding that appears consistent across both MVs and TVs. Validation in larger cohorts will be necessary to confirm this observation. Although our method allows the study of leaflet strain progression over time and the evaluation of postoperative leaflet strain, it cannot be used to predict surgical outcomes in advance without a target systolic surface. Research efforts aimed at improving the fidelity of FEA are critical to accurately emulate ground-truth physiological valvular dynamics and enable *de novo* forward simulations to identify the optimal repair in an individual patient.

## 5 Conclusions

We have developed a novel point-based registration approach for tracking leaflet motions across the diastole and systole frames. Our method provides a comparable strain estimation to that in FEA. Our approach was successfully applied to three groups of atrioventricular valves across pediatric and adult patients. The cohorts encompassed a broad spectrum of valve anatomy, from healthy mitral valves to those with restricted leaflets and pronounced billowing. Although we did not identify a unifying strain pattern that clearly distinguishes healthy from diseased valves, we observed a promising trend in which a higher median and IQR of strain distribution appears to be a strong marker of leaflet billowing. With further validation, this metric could serve as a novel quantitative tool to enhance prognostication of valvular disease.
